# Absence of meningeal mast cells in the *Mitf* mutant mouse

**DOI:** 10.3389/fncel.2024.1337621

**Published:** 2024-02-09

**Authors:** Alba Sabaté San José, Petur Henry Petersen

**Affiliations:** ^1^Department of Anatomy, Biomedical Center, Faculty of Medicine, University of Iceland, Reykjavik, Iceland; ^2^ULB Neuroscience Institute (UNI), Université Libre de Bruxelles (ULB), Brussels, Belgium

**Keywords:** microphthalmia-associated transcription factor, mast cells, meninges, inflammation, aging

## Abstract

Mast cells (MCs) are located in the meninges of the central nervous system (CNS), where they play key roles in the immune response. MC-deficient mice are advantageous in delineating the role of MCs in the immune response *in vivo*. In this study, we illustrate that a mutation in microphthalmia-associated transcription factor (*Mitf*) affects meningeal MC number in a dosage-dependent manner. C57BL/6J *Mitf* null mice lack meningeal MCs completely, whereas heterozygous mice have on average 25% fewer MCs. *Mitf* heterozygous mice might be a valuable tool to study the role of MCs in the meninges.

## 1 Introduction

Mast cells (MCs) are tissue-resident immune cells found throughout the body, especially at the interface between the body and the external environment, where they take part in the earliest immune responses. The immune responses of the central nervous system (CNS) are restricted in comparison to the rest of the body due to the presence of the blood–brain barrier (BBB), which serves as an internal boundary separating the CNS from the body. MCs are located at the BBB (Khalil et al., 2007) but also in the protective tissues surrounding the CNS, the meninges. The meninges are divided into three layers: dura, arachnoid, and pia. Arteries lie in the subarachnoid space, and MCs are located primarily in the dura in linear arrays alongside the arteries (Dimlich et al., 1991) and in proximity to neurons, including nociceptors (Rozniecki et al., 1999). MCs likely play a role in the normal physiological function of the meninges and are important for maintaining the BBB (Sayed et al., 2010). When activated, MCs degranulate a multitude of bioactive substances (including biogenic amines and MC-specific proteases), which affect other cells. MCs have different secretive phenotypes depending on the tissue microenvironment (Gurish and Austen, 2012). They are best known for their role in allergies as first responders to potentially harmful substances, but their role in the immune response following infections is still not fully understood (Valent et al., 2020; Dahlin et al., 2022). Meningeal MCs have also been shown to play roles in neurogenic inflammation, i.e., inflammation without infection, a central mechanism in migraines (Levy et al., 2006, 2019). Additionally, meningeal MCs are implicated in the outcome of stroke and other CNS pathologies (Arac et al., 2014; Russi et al., 2018). Understanding the role of MCs in the meninges is therefore of high importance.

### 1.1 The role of meningeal mast cell and mouse models

Inflammation is a central mechanism in CNS pathology with both short- and long-term effects. Inflammation can occur without infection, become chronic, or increase with age (inflammaging). Understanding how inflammatory processes can be influenced negatively or positively is instrumental in alleviating challenging pathological conditions found in many neurological diseases. During pathological conditions, crosstalk between different cell types is complex, especially in the CNS, and it is therefore difficult to model cell–cell interactions, e.g., in cell cultures. More complex models are needed to understand the role of MCs in the immune response, and mouse models that lack meningeal MCs have been central in delineating their role in the CNS. Many of these studies are based on mutations in Kit/Kitl—genes encoding for a growth factor and its receptor important for MC progenitor proliferation and differentiation (Grimbaldeston et al., 2005). Another mouse model is mutations in the microphthalmia-associated transcription factor (*Mitf*), which results in a stark reduction in MC number or absence depending on tissue (Kitamura et al., 2002; Ingason et al., 2019). There are other models in which MCs are targeted for apoptosis or depleted (Reber et al., 2012; Feyerabend et al., 2016; Sasaki et al., 2021; Dahlin et al., 2022).

### 1.2 The *Mitf* gene is required for mast cell generation and function

MITF is a basic helix–loop–helix leucine zipper transcription factor that plays a critical role in the development of many cell types (Hemesath et al., 1994; Steingrimsson et al., 2004), including melanocytes, retinal pigment cells, osteoclasts, and a subpopulation of CNS neurons (Nakayama et al., 1998; Steingrimsson et al., 2002; Gudjohnsen et al., 2015; Atacho et al., 2020). The *Mitf* gene has been shown to be both important for the generation (Kitamura et al., 2002; Morii et al., 2004; Sasaki et al., 2016; Ingason et al., 2019) and function of MCs (Morii et al., 1996, 1997; Nechushtan and Razin, 2002; Morii and Oboki, 2004; Paruchuru et al., 2022). There are connections between *Mitf* and Kit signaling (Tsujimura et al., 1996; Lee et al., 2011), and *Mitf* plays a key role in the binary lineage choice between MCs and basophils during development. The bipotent basophil/MC progenitors (BMCPs) develop into basophils or MCs depending on the relative ratio of C/EBPα to MITF, which are negatively correlated with each other (Qi et al., 2013; Sasaki et al., 2016). *Mitf* mutations cause these progenitors to develop into basophil-like cells. In *Mitf* null mutant mice, MCs are absent from the peritoneum (Morii and Oboki, 2004) and the heart (Ingason et al., 2019) and are reduced in number in the skin (Kim et al., 1999; Morii and Oboki, 2004). To date, the effect of the loss of *Mitf* on meningeal MCs has not been reported.

## 2 Methods

### 2.1 Animals

Animals of both sexes were used from the following mouse strains: C57BL/6 J (wild type) and *Mitf* mutant mice: heterozygote C57BL/6 J-*Mi^-mi-vga9/+^* and homozygote C57BL/6 J-*Mitf^-mi-vga9/−mi-vga9^*. All animals were kept at the animal facility of the University of Iceland (VRIII) in a controlled environment at 21–22°C. Food and water were provided *ad libitum*. Sacrifice was performed by cervical dislocation. Animal procedures were approved by the Committee on Experimental Animals, according to Regulation 460/2017 and European Union Directive 2010/63 (license number 2013-03-01).

### 2.2 Avidin staining

Following euthanasia, the calvarium was removed from the skull, and the meninges adherent to the calvarium were fixed in 4% PFA for 30 min and rinsed with cold PBS. The whole meninges were removed, permeabilized, and blocked with blocking buffer (0.1% Triton 100x and 5% normal goat serum in PBS) for 1 h at room temperature. The samples were incubated with avidin AF488 conjugated (Catalog number A21370, Invitrogen) diluted 1/1000 and DAPI in a blocking buffer for 1 h at room temperature (Tharp et al., 1985). Finally, the samples were washed three times with 0.1% Triton X-100 in PBS for 20 min and mounted on slides using Fluoromount. Images of the whole meninges were acquired using an Olympus FV10-MCPSU Confocal Microscope.

### 2.3 Toluidine blue staining

The meninges were stained attached to the calvarium. The samples were submerged in a toluidine blue staining solution (1 g toluidine blue/100 mL of 70% EtOH in dH2O) for 5 min at room temperature. Subsequently, the meninges were washed three times in distilled H_2_O for 5 min, rinsed five times in 90% EtOH, and five times in 96% EtOH. Finally, the meninges were dissected from the calvarium and mounted on slides using solidifying mounting media. A Leica DM LB microscope (Leica Microsystems) at 10x magnification was used for image acquisition.

### 2.4 Statistical methods

The results obtained from MC quantification were analyzed by unpaired Student’s t-tests using GraphPad Prism 9. Numerical results represent the mean amount of MC/mm^2^ and the standard error of mean (SEM).

## 3 Results

### 3.1 Dosage dependence of *Mitf* on meningeal mast cells/results

**Figure 1:**
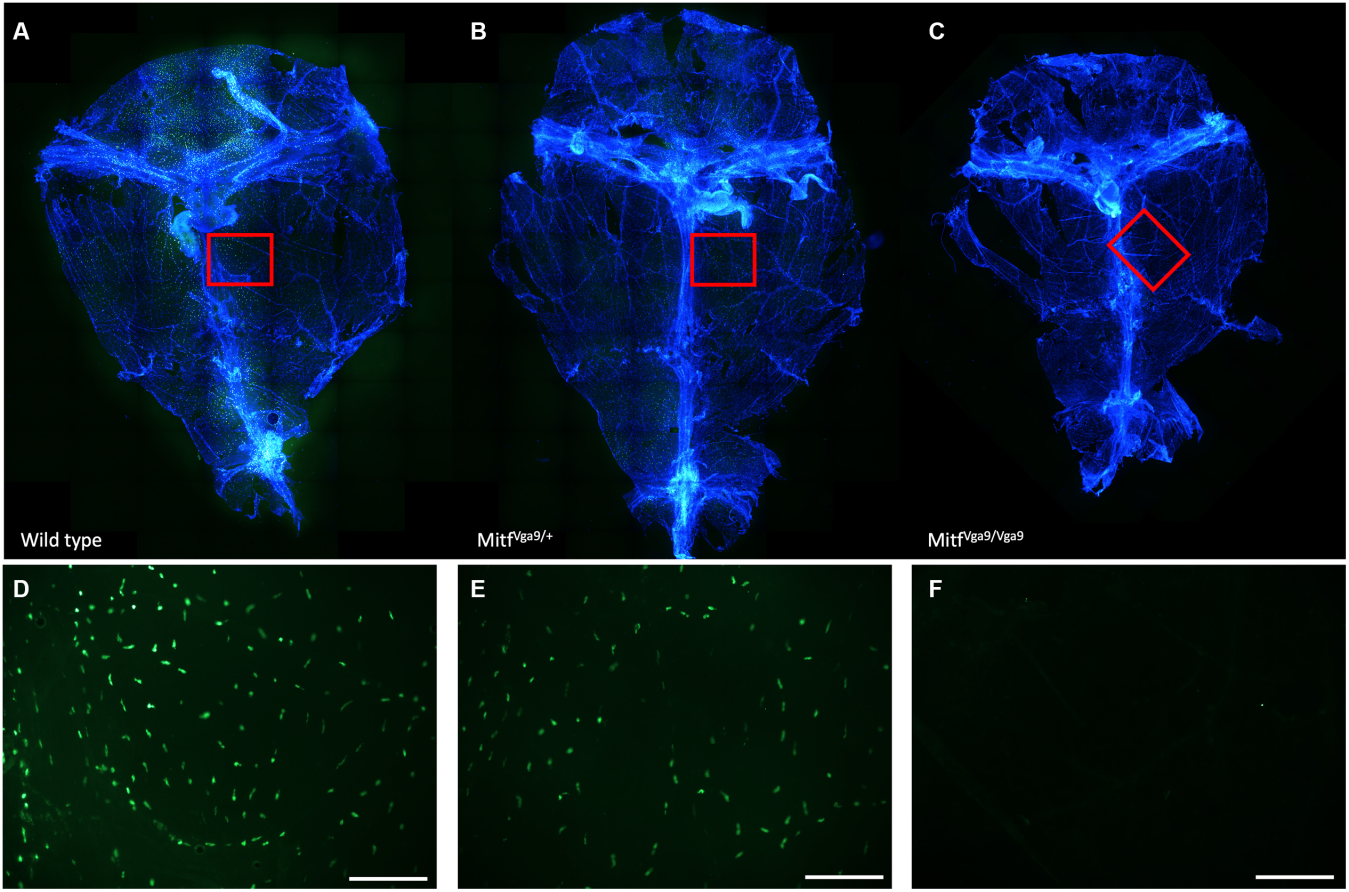
Distribution of MCs within meninges. Avidin staining of whole meninges of wild-type **(A)**, *Mitf* heterozygote [**(B)** *Mitf^mi-vga9/+^*], and *Mitf* homozygote [**(C)** *Mitf^mi-vga9/mi-vga9^*] mice. Below **(D–F)** is a close-up of the meningeal MC distribution.

**Figure 2:**
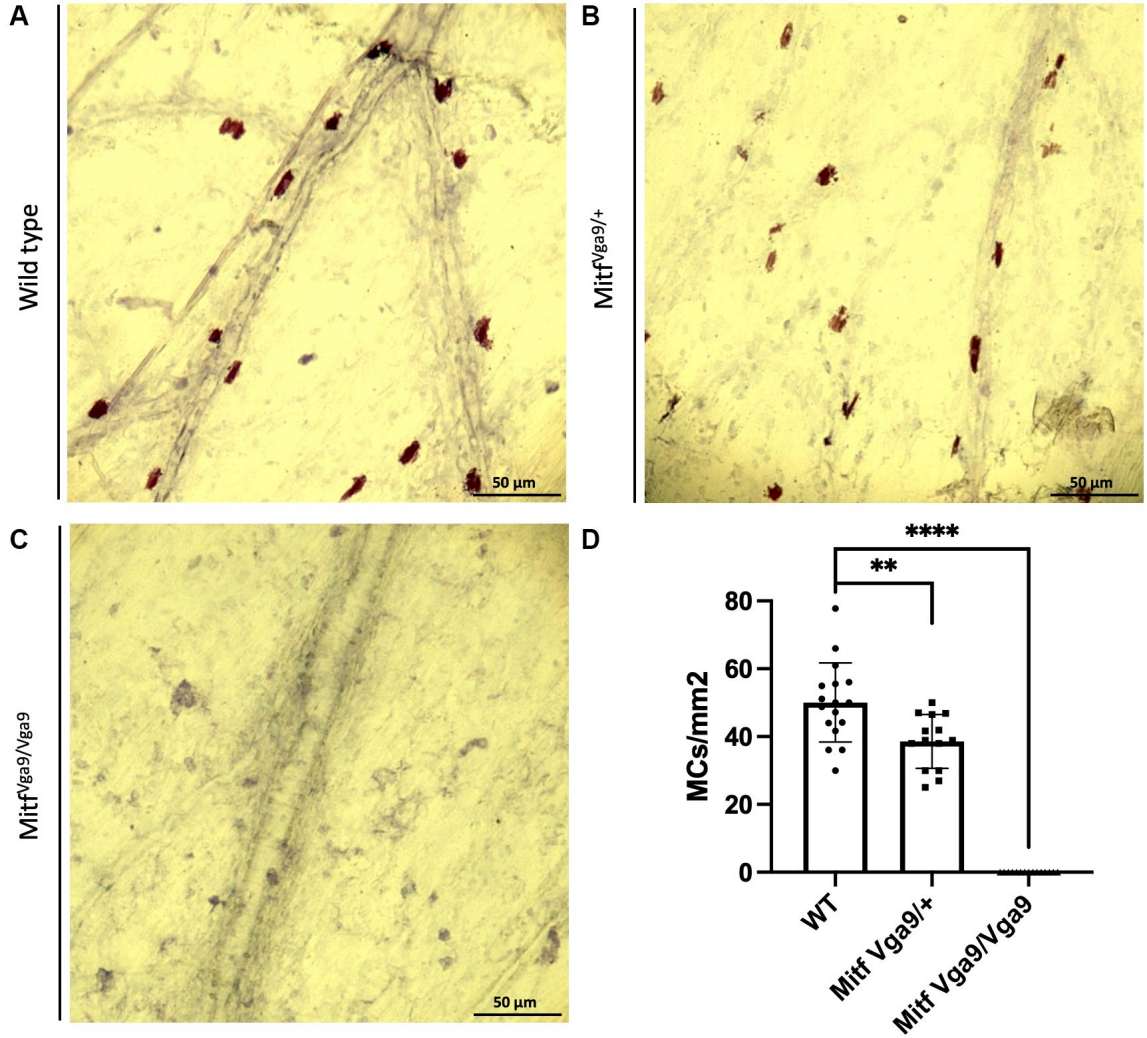
Loss of *Mitf* leads to a reduction in meningeal MC number. Toluidine blue staining of MCs from wild-type **(A)**, *Mitf* heterozygote [**(B)** *Mitf^mi-vga9/+^*], and *Mitf* homozygote [**(C)** *Mitf^mi-vga9/mi-vga9^*] mouse meninges. **(D)** MC number in different genotypes. Wild-type mice *n* = 17, heterozygote C57BL/6 J-*Mitf^mi-vga9/+^ n* = 14, C57BL/6 J-*Mitf^mi-vga9/mi-vga9^ n* = 15. Asterisks indicate statistical significance [*p* < 0.01 (**) and *p* < 0.0001 (****)]. Scale bar represents 50 μm.

To determine the degree to which meningeal MCs are affected by the lack of *Mitf*, the dura was collected from wild-type, heterozygous, and mutant adult mice, 2–3 months old. MCs were stained with avidin, a glycoprotein that binds to the granules of MCs (Tharp et al., 1985; Figure 1). There was a clear absence of MCs in the dura of the mutant animals and a reduction in MC number in heterozygotes. To quantify the number of MCs, toluidine blue, a dye that stains specifically acidic tissue components, and MC granules in purple were used to identify MCs in meninges. Toluidine staining of dura MCs confirmed the reduction of MC number in the heterozygotes (41.04 /mm^2^ ± 2.23 *n* = 14, wild type 49.9/mm^2^ ± 2.23 *n* = 17), while in the mutant no MCs (*n* = 15) were detected (Figure 2). No difference was observed between the sexes (*N* = 10, heterozygotes *p* = 0.288, wild type *p* = 0.642). The reduction in the heterozygotes was on average 25%, and not all heterozygotes showed a reduction. Some variation in the difference was observed between different experiments (difference between wild-type and heterozygotes: 10–28%).

## 4 Discussion

MCs play a central role in the meninges and have diverse connections to CNS pathology, especially inflammation but also stroke, migraine, and neurological diseases. Mouse models of MC deficiency are crucial to the study of MC biology, which is often difficult to address in *ex vivo* models (human material or cell culture) due to cell–cell interactions. Of these models, the *Mitf* mutant mouse is promising due to the reduction of MCs throughout the body (Morii et al., 2004; Ingason et al., 2019). We show here that *Mitf* null mice lack meningeal MCs and can therefore be used to study the role of MCs in the immune responses defending the CNS. However, it is important to note that studies using constitutive *Mitf* mutant mice must also consider the lack of meningeal melanocytes (Gudjohnsen et al., 2015) and that any hypotheses of *Mitf* function in the CNS must take the lack of both of these cell types into account.

Importantly, *Mitf* heterozygous mice, which are phenotypically identical to wild-type mice, show a reduction in meningeal MC number as previously reported for cardiac MCs (Ingason et al., 2019). In the heart, the reduction of MCs in the heterozygote is approximately 50% (Ingason et al., 2019) and in the meninges, it is approximately 25%. While two copies of *Mitf* are required for a normal number of MCs, this suggests a tissue-dependent *Mitf* dosage effect or defects in maintaining MCs in heterozygotes. This is in accordance with known haploinsufficiency in *Mitf* mutant mice (Harris et al., 2018; Atacho et al., 2020; Han et al., 2020). Increased inflammation is central to CNS pathologies. Aging affects MCs (Gunin et al., 2011; Chatterjee and Gashev, 2012; Kutukova et al., 2016) and has been reported to be proinflammatory (Elbasiony et al., 2023) during aging. As *Mitf* is also required for MC function, *Mitf* heterozygous mice might be more applicable for studies of meningeal MCs during aging in comparison to other MC models, which are limited due to the absence or reduced number of MCs. Further studies are needed to examine the extent of this effect and the possibility of using *Mitf* heterozygotes to further explore the compromised function of meningeal MCs during aging.

## Data availability statement

The raw data supporting the conclusions of this article will be made available by the authors, without undue reservation.

## Ethics statement

The animal study was approved by the Icelandic Committee on Experimental Animals. The study was conducted in accordance with the local legislation and institutional requirements.

## Author contributions

AS: Data curation, Investigation, Writing – review & editing. PP: Conceptualization, Investigation, Methodology, Project administration, Writing – original draft, Writing – review & editing.
